# Proerythroblast Cells of Diamond-Blackfan Anemia Patients With *RPS19* and *CECR1* Mutations Have Similar Transcriptomic Signature

**DOI:** 10.3389/fphys.2021.679919

**Published:** 2021-06-11

**Authors:** Beren Karaosmanoglu, M. Alper Kursunel, Duygu Uckan Cetinkaya, Fatma Gumruk, Gunes Esendagli, Sule Unal, Ekim Z. Taskiran

**Affiliations:** ^1^Department of Medical Genetics, Faculty of Medicine, Hacettepe University, Ankara, Turkey; ^2^Department of Stem Cell Sciences, Institute of Health Sciences, Hacettepe University, Ankara, Turkey; ^3^Department of Basic Oncology, Cancer Institute, Hacettepe University, Ankara, Turkey; ^4^Division of Pediatric Hematology, Department of Pediatrics, Faculty of Medicine, Hacettepe University, Ankara, Turkey; ^5^Center for Stem Cell Research and Development, Hacettepe University, Ankara, Turkey; ^6^Research Center for Fanconi Anemia and Other IBMFS, Hacettepe University, Ankara, Turkey

**Keywords:** Diamond Blackfan Anemia, ribosomopathy, proerythroblast, *RPS19*, *CECR1*, transcriptomics

## Abstract

Diamond Blackfan Anemia (DBA) is an inherited bone marrow (BM) failure syndrome, characterized by a paucity of erythroid differentiation. DBA is mainly caused by the mutations in ribosomal protein genes, hence classified as ribosomopathy. However, in approximately 30% of patients, the molecular etiology cannot be discovered. *RPS19* germline mutations caused 25% of the cases. On the other hand, *CECR1* mutations also cause phenotypes similar to DBA but not being a ribosomopathy. Due to the blockade of erythropoiesis in the BM, we investigated the transcriptomic profile of three different cell types of BM resident cells of DBA patients and compared them with healthy donors. From BM aspirates BM mononuclear cells (MNCs) were isolated and hematopoietic stem cells (HSC) [CD71^–^CD34^+^ CD38^mo/lo^], megakaryocyte–erythroid progenitor cells (MEP) [CD71^–^CD34^+^ CD38^hi^] and Proerythroblasts [CD71^+^ CD117^+^ CD38^+^] were sorted and analyzed with a transcriptomic approach. Among all these cells, proerythroblasts had the most different transcriptomic profile. The genes associated with cellular stress/immune responses were increased and some of the transcription factors that play a role in erythroid differentiation had altered expression in DBA proerythroblasts. We also showed that gene expression levels of ribosomal proteins were decreased in DBA proerythroblasts. In addition to these, colony formation assay (CFU-E) provided functional evidence of the failure of erythroid differentiation in DBA patients. According to our findings that all patients resembling both *RPS19* and *CECR1* mutations have common transcriptomic signatures, it may be possible that inflammatory BM niche may have a role in DBA pathogenesis.

## Introduction

Diamond Blackfan Anemia (DBA) (OMIM# 105650) is a rare (5–10/1,000,000) bone marrow (BM) failure syndrome, characterized by blockade in erythropoiesis at earlier stages (Da Costa et al., 2018) and usually affects only erythroid lineage cells in the BM, however, pancytopenia may also be seen in rare cases (Garçon et al., 2013; Danilova and Gazda, 2015). Patients usually have normochromic and macrocytic, severe anemia with reticulocytopenia. DBA is usually inherited autosomal dominantly (40%), due to loss of function mutations or deletions in genes encoding ribosomal proteins. On the other hand, most of the other patients have *de novo* sporadic mutations. Approximately 50% of the patients have physical anomalies like craniofacial defects, renal anomalies, limb anomalies, and cardiac defects (Jaako et al., 2014; Nakhoul et al., 2014). The mutations on the genes that encode ribosomal proteins are found to be linked with DBA, however, molecular etiology cannot be clarified in approximately 30% of the patients. To date, 19 of 79 ribosomal protein genes were associated with DBA (*RPS19*, *RPL5*, *RPS26*, *RPL35A*, *RPL11*, *RPS1*, *RPS24*, *RPS17*, *RPS7*, *RPL26*, *RPS29*, *RPL15*, *RPS28*, *RPL31*, *RPS27*, *RPL27*, *RPL35*, *RPL18*, and *RPS15A*) (Avondo et al., 2009; Aspesi et al., 2014; Pereboom et al., 2014; Kattamis, 2020). Due to the mutations in genes encoding ribosomal proteins, DBA is classified as a ribosomopathy, which is a failure in ribosome biogenesis. According to the previous studies, 25% of the cases are associated with germline mutations in *RPS19* ribosomal protein (Gazda et al., 2006; Garçon et al., 2013). On the other hand, recently, *GATA-1* and *TSR2* mutations have been shown to cause DBA phenotype through X-linked inheritance pattern (Sankaran et al., 2012; Gripp et al., 2014).

In recent studies, it has been noted that *CECR1* mutations can also cause a DBA-like phenotype. Although *CECR1* mutations are known as DADA2 deficiency, which causes a wide range of phenotypes such as intermittent fevers, stroke, and polyarteritis nodosa, it is not yet known by which mechanism it causes DBA-like phenotype (Sasa et al., 2015; Ulirsch et al., 2018).

In the present study, BM resident hematopoietic stem/progenitor cells from both DBA patients (both *RPS19* and *CECR1* mutations) and healthy donors were analyzed with a transcriptomic approach. We identified some gene expression differences related to cellular response to stress in DBA proerythroblasts.

## Materials and Methods

### Patients

Patients included in this study were diagnosed with DBA according to the established criteria (Vlachos et al., 2008). Healthy BM transplantation donors were chosen as age and gender matching with DBA patients, from the Hacettepe University Department of Pediatrics BM Transplantation Unit and PEDI-STEM (Center for Stem Cell Research and Development) BM registry. BM aspirate materials from 3 DBA patients and 4 healthy donors were included in this study. The clinical information and genotypic details of the individuals were given in Table 1. The peripheral blood complete blood count analyses and the BM samples were obtained on the transfusion day and before transfusion was made. All of the clinically diagnosed DBA patients were first screened for *RPS19* point and copy number mutations. Genome-wide copy number analysis and whole-exome sequencing were performed for the patient without any *RPS19* variation. Genomic studies were completed within The European Diamond-Blackfan Anemia Consortium (EuroDBA). The study protocol was approved by the Hacettepe University Local Ethical Committee (GO 15/721-19), according to the ethical standards of the Declaration of Helsinki.

**TABLE 1:** Clinical information of DBA patients.

Sample code	DBA-1	DBA-2	DBA-3
Mutation	*RPS19* (p.Tyr79*)	*RPS19* (p.Gln11*)	*CECR1* (p.Tyr227fs*27)
Diagnosis age (month)	1	1	8
BMA age (year)	6	1	3
Gender	M	M	F
MCV (fl)	105.8	85.6	89.9
Erythrocyte count (10^9^/ul)	3,180	2,800	4,470
Hemoglobin (gr/dL)	11.2	8.6	12.9
Treatment on the day of BMA	Methylprednisolone (0.25 mg/kg)	Hydrocortisone (10 mg/m^2^); deferasirox (12 mg/kg), on transfusion program	On transfusion program

*The values of the hematological profiles of DBA-2 and DBA-3 were measured as pre-transfusion. BMA: bone marrow aspiration, MCV: mean corpuscular volume, fl: femtoliter, M: male, F: female.*

### Mononuclear Cell Isolation From BM Aspirate

Mononuclear cells (MNCs) were isolated from BM aspirate according to phase separation method with Ficoll^TM^ and frozen at −150°C for further studies. After all samples were collected, BM MNCs were thawed with DMEM (supplemented with 10% FBS, 1% penicillin-streptomycin, and 1% l-glutamine) and immediately taken to cell sorting procedure or thawed with thawing medium for colony forming assay.

### Erythroid Colony-Forming Assay (CFU-E)

Bone marrow-MNCs were thawed in 10 ml thawing medium consisting of PBS, FBS (2%), and DNase I (10 ug/ml, DN25, Sigma). 150.000 cells were suspended in IMDM (Gibco), mixed with 1 ml Methocult with EPO (H4330; Stem Cell Technologies, Vancouver, Canada), which was supplemented with Hemin (0.002 M, 51280-1G, Sigma) and Stem Cell Factor (SCF, 0.2 mg/ml, 573904, Biolegend). Semisolid medium-cell mix was plated in 35 mm petri dishes with blunt end needle in duplicate. After 14 days, colony numbers were counted.

### Immunophenotyping and Cell Sorting With Fluorescence-Activated Cell Sorting

The antibodies; anti-human-CD34 (8G12), -CD117 (104D2) (BD Biosciences), -CD38 (HIT2), -CD71 (CY1G4) (Biolegend), -eFluor660 F(ab’)2 (eBiosciences) were used in immunophenotyping analyses and fluorescence-activated cell sorting (FACS)-based cell sorting. The percentage of positive cells were determined in comparison with the isotype-matched antibody controls. Immunophenotyping and cell sorting were performed from the BM MNCs of healthy donors and DBA patients with BD FACSAria^TM^ II (BD Biosciences). Surface markers were chosen as previously described (Chen et al., 2009; Attar, 2014). Accordingly, hematopoietic stem cells (HSC) were gated as CD71^–^CD34^+^CD38^mo/lo^, Megakaryocyte–erythroid progenitor cells (MEP) were gated as CD71^–^CD34^+^CD38^hi^, Proerythroblasts (Pro-E) were gated as CD71^+^ CD117^+^ CD38^+^, Basophilic Erythroblasts (Baso-E) were gated as CD71^+^ CD117^–^CD38^+^ and Polychromatophilic Erythroblasts (Poly-E) were gated as CD71^+^ CD117^–^CD38^–^ populations (Supplementary Figure 1). Healthy donor MNCs were pooled before cell sorting. Cell percentages of all samples were given in Supplementary Figure 2. Four different cell populations (HSC, MEP, proerythroblasts, and Baso/Poly erythroblasts) were sorted, 3 cell types (HSC, MEP, and proerythroblasts) were taken to transcriptomic profiling. Because Baso/Poly erythroblasts were heterogeneous, transcriptomic profiling was not performed.

### RNA Isolation, Library Preparation, and Sequencing

After cell sorting, RNA was isolated with Single Cell RNA Purification Kit (Norgen Biotek Corp.) and stored at −80°C for further experiments. Total RNA samples of HSC, MEP, and proerythroblasts were amplified with REPLI-g WTA Single Cell Kit (Qiagen). Transcriptome libraries were prepared with Ion AmpliSeq^TM^ Transcriptome Human Gene Expression Kit (Thermo Fisher Scientific) on Ion Chef Instrument (Thermo Fisher Scientific). Library quantitation was measured with Qubit dsDNA High Sensitive Quantitation Kit by using Qubit 2.0 Instrument (Thermo Fisher Scientific). Combined libraries were clonally amplified by emulsion PCR and Ion PI Hi-Q OT2 200 Kit was used. Next-generation sequencing (NGS) reaction was performed with Ion PI Hi-Q Sequencing 200 Kit on Ion Proton Semiconductor Sequencer (Thermo Fisher Scientific).

### Data Analysis

For quantitative transcriptomic analysis, plug-in codes in Ion Torrent server were used. After NGS reaction, raw reads were normalized according to the reads per million (RPM) method and mapped to genome assembly hg19 AmpliSeq Transcriptome version by TMAP (Torrent Mapping Alignment Program). Each sample was studied in duplicates. Sequence read counts were given in Supplementary Table 1. For differentially expressed gene (DEG) analysis DESeq2 codes were used. The gene expression differences with a fold change higher than 2 and FDR cutoff value was lower than 0.05 were considered as significant. iDEP.91 bioinformatic analysis tool was used for further analysis and data visualization^1^ (Ge et al., 2018). For functional enrichment analysis, g:Profiler was used^2^ by using g:SCS (set counts and sizes) correction method with default parameters (Raudvere et al., 2019). Ordinary one-way ANOVA was used was performed using GraphPad Prism version 8.4.3. for Windows (GraphPad Software, La Jolla, CA, United States)^3^. Expression data have been submitted to the Dryad Digital Repository^4^.

## Results

### Diamond Blackfan Anemia Patient Samples Demonstrate Diminished/Lack of Colony-Forming Potential

**FIGURE 1:**
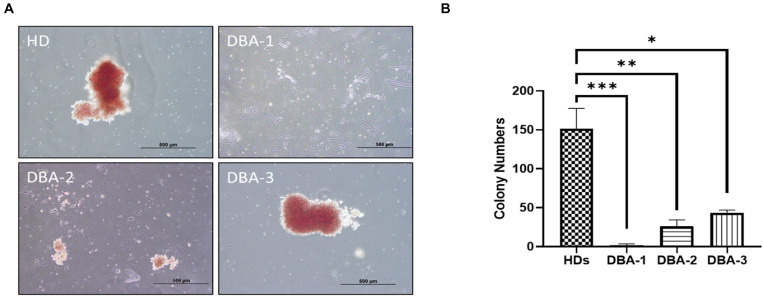
Erythroid colony formation assay. **(A)** DBA patient samples had diminished/lack of colony forming potential **(B)** and had significantly low number of erythroid colonies. HD: Healthy donor (^∗^*p*: 0.0015; ^∗∗^*p*: 0.0007; ^∗∗∗^*p*: 0.0003).

After 14 days of incubation, samples were examined for their erythroid colony formation capacity. All healthy donor samples successfully formed colonies. The sample of DBA-1 did not form any colonies, samples of DBA-2 and DBA-3 showed lower colony-forming potential (Figure 1). Interestingly, the morphology and size of the colonies of DBA-3 were similar to the healthy donors’. But generally, DBA patient samples had a failure at the formation of mature colonies and formed significantly low colony numbers. At this stage, only colonies of DBA-3 were taken into transcriptomic profiling due to the colony presence. However, there were no significant gene expression differences when compared with healthy donor colonies (data not shown).

### Diamond Blackfan Anemia Proerythroblasts Had a Distinct Transcriptomic Profile

**FIGURE 2:**
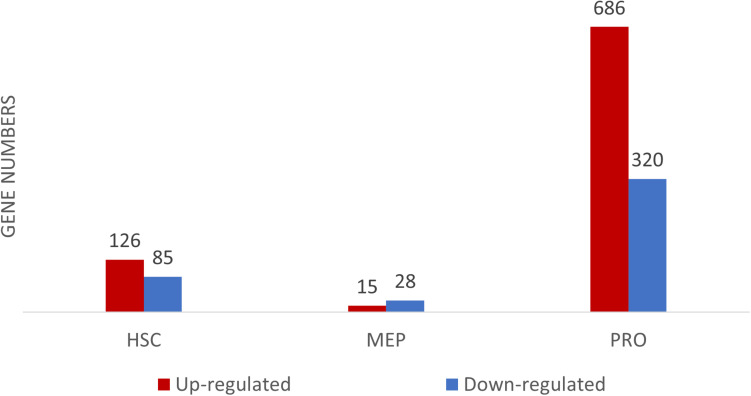
Differentially expressed gene (DEG) numbers between DBA and healthy donors. For DEG analysis, all three DBA samples were grouped and compared with healthy donors.

The transcriptomic profiles of some of the BM stem/progenitor cells (HSC, MEP, and proerythroblasts) were examined. The highest number of DEGs, between DBA patients and healthy donors, were observed in proerythroblasts (686 up-regulated, 320 down-regulated genes) (Figure 2 and Supplementary Table 2a). According to the enrichment analysis (g:Profiler), there were no significant pathways found in HSCs and MEPs (Supplementary Figure 3). However, it was striking that there were many differentially expressed immune system-related genes in HSCs (Supplementary Figure 4 and Supplementary Table 2b). The total number of DEGs of MEPs was low (43 transcripts), interestingly, increased expression of *CDKN1A* (p21) was remarkable (Supplementary Table 2c).

**FIGURE 3:**
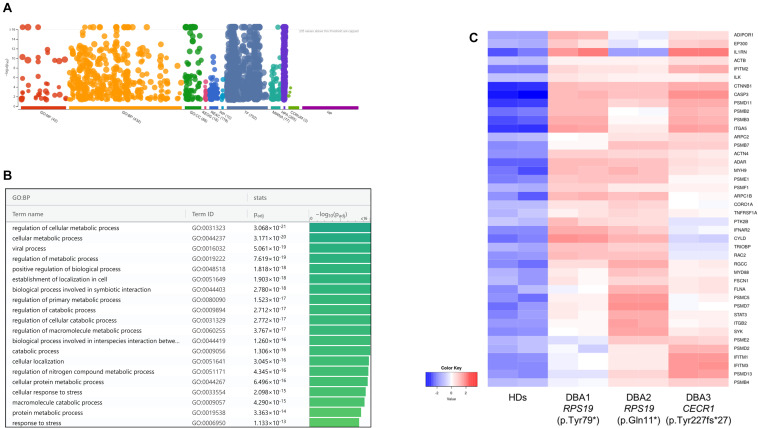
**(A)** Pathway analysis of the upregulated genes, **(B)** significant pathways belong to biological process (BP) performed using g:Profiler, **(C)** differentially expressed genes belong to cellular stress in DBA proerythroblasts. BP: Biological Process, HDs: healthy donors.

According to the pathway enrichment analysis, only up-regulated genes of DBA proerythroblasts showed significant results (Figure 3, Supplementary Figure 3, and Supplementary Table 3). Most of the up-regulated genes were mainly responsible for cellular metabolic processes. Among up-regulated pathways in DBA proerythroblasts, the cellular response to stress (GO:0033554) pathway was of notice (*p*-value: 6.891e-16). Among these, cytokine response and proteasome related genes were striking (Figure 3C).

At this stage, it was observed that there was a common gene expression profile between patients resembling *CECR1* and *RPS19* mutations, compared to healthy donors (Supplementary Figure 5 and Supplementary Table 4). When examining patient-specific gene expression changes in detail, it was noteworthy that transcripts such as *NFKB1*, *IL1B*, *NFKBIZ*, and *NFKBID* were up-regulated in individual with homozygous *CECR1* mutation. On the other hand, no significant patterns were found for DEGs specific to individuals with *RPS19* mutations. Hence, regardless of the genotype, it was thought that there could be a common DBA-specific transcriptional response in proerythroblasts.

### Gene Expression Levels of Ribosomal Proteins Were Decreased in DBA Proerythroblasts

**FIGURE 4:**
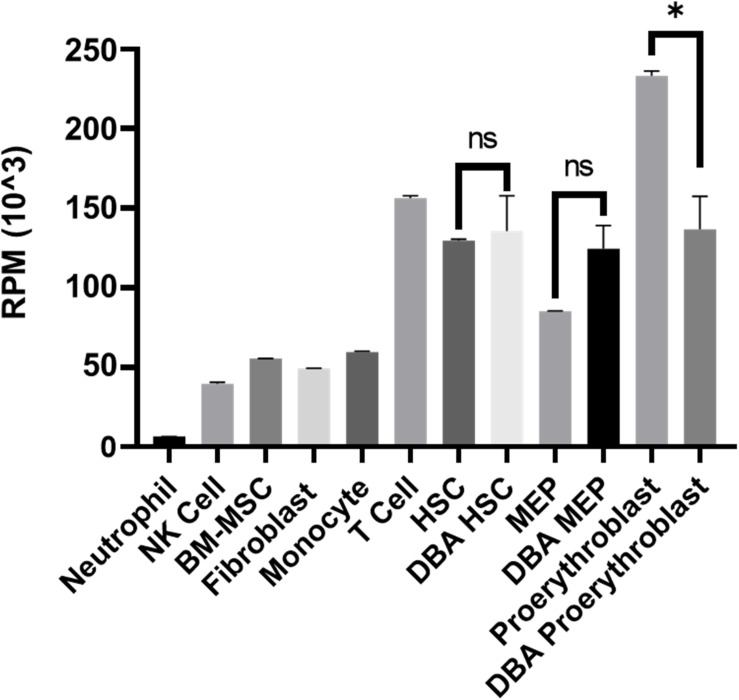
Total ribosomal protein mRNA expression of different cell types. Different cell types analyzed with the same transcriptomic approach from the in-house dataset were evaluated. RPM: reads per million, NK: natural killer, BM-MSC: bone marrow mesenchymal stem cells. HSC: hematopoietic stem cell, MEP: Megakaryocyte–Erythroid Progenitor Cell, ns: non-significant. ^∗^*p* value < 0.0001.

Although there was no significant pathway enrichment for decreased transcripts of proerythroblasts (Supplementary Figure 3), reduced expression of ribosomal protein genes was striking. Therefore, total transcriptomic data was evaluated in terms of all 75 ribosomal protein-coding genes. In DBA proerythroblasts, total ribosomal protein gene expression was significantly lower than the healthy donors. Furthermore, according to our in-house data, which was generated with the same NGS library preparation method and sequencing platform, proerythroblasts had the highest total ribosomal protein gene expression level (Figure 4). Total ribosomal protein gene expression levels of HSCs and MEPs did not show a significant difference. Interestingly, the patient with *CECR1* mutation (DBA-3), also showed low total ribosomal protein expression as the other patients with harboring *RPS19* mutation (Supplementary Figure 6). In addition to the total reduction of ribosomal protein expression levels, *BAZ2A*, which is responsible for repressing RNA polymerase I transcription, was found to be the most increased transcript (log2 fold change: 6.28) in DBA proerythroblasts.

### Diamond Blackfan Anemia Proerythroblasts Have Altered Transcription Factor mRNA Expression

**FIGURE 5:**
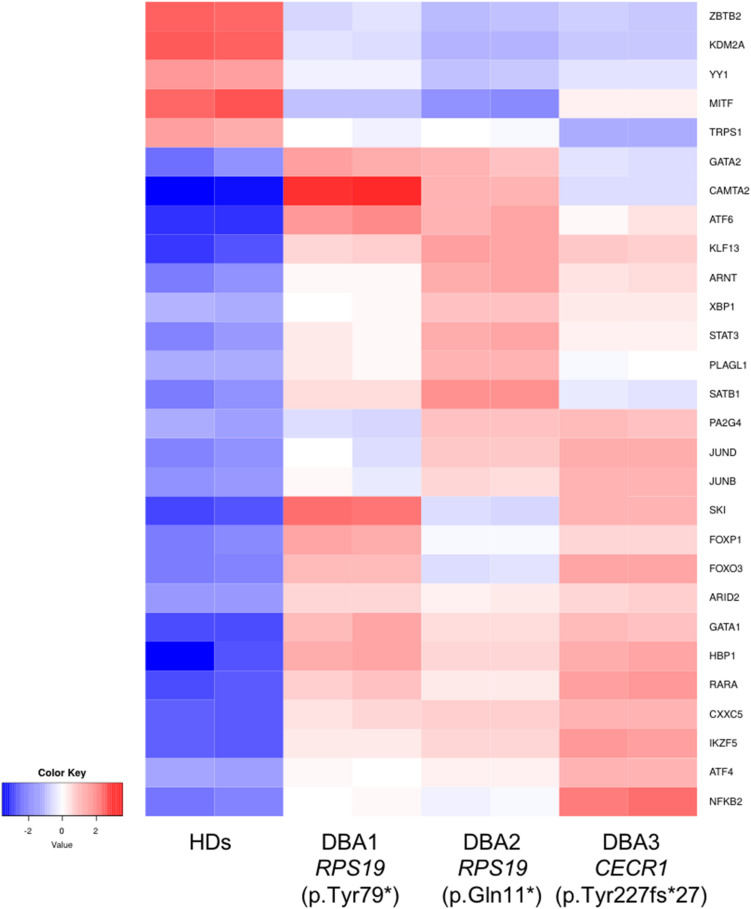
Differentially expressed transcription factors that are primarily related with erythroid lineage or other critical cellular responses. HDs: healthy donors.

In order to identify key gene expression regulators in DBA proerythroblasts, we examined all known transcription factors (nearly 1,600) and 76 of them were found to be altered (Supplementary Table 5). Among these, the expression levels of some of the critical transcription factors that negatively/positively regulates erythroid differentiation (*ARNT*, *FOXO3*, *GATA1, GATA2*, *KLF13*, *SATB1*, *SKI*, *STAT3*, and *TRPS1*) were striking (Figure 5). In addition, other transcription factors that could be related to cellular stress or inflammation were also of notice (Figure 5).

## Discussion

Identification of the molecular signature and genotype-phenotype correlation of rare syndromes is an important step toward understanding disease mechanisms and developing potential therapeutics. For many years, the molecular pathophysiology of DBA was investigated in different *in vitro* and *in vivo* models. DBA is a rare (5–10/1,000,000) BM failure syndrome with a paucity of erythroid progenitor cells. To date, different groups performed microarray-based transcriptomic studies of BM -derived, peripheral blood-derived cells, or fibroblast cells of DBA patients (Gazda et al., 2006; Koga et al., 2006; Avondo et al., 2009; O’Brien et al., 2017). Gazda et al. (2006) determined that the pathways related to apoptosis and cancer had altered in adult DBA patients in remission at the time. O’Brien et al. (2017) studied with relatively younger (transfusion or steroid-dependent) patients with RP and *GATA1* mutations, and they demonstrated that peripheral blood-derived CD34 ^+^ cells showed altered gene expression profile. The strength of this work is the comparison of the DBA and DADA2 transcriptional profiles. It has been a question in the field as to why a subset of DADA2 patients has a phenotype that overlaps with that of DBA, but that DBA patients rarely have the fever/inflammation symptoms typically seen in DADA2 patients. Here, we purified BM resident progenitor cells of young DBA patients and analyzed their transcriptomic profile.

According to our transcriptomic profiling results, the most drastic change was observed in the gene expression profile of DBA proerythroblasts. It was remarkable that the expression of the genes associated with cellular stress was increased and some of the transcription factors that play a role in erythroid differentiation had altered expression. Some of the genes associated with the cellular stress response were also associated with the immune response. In addition, the presence of some changes in immune system-related genes in the DBA HSCs may be another evidence of increased inflammation in the BM microenvironment. Although minimal changes were detected at the transcriptomic level, the *CDKN1A* expression increase in MEPs should be validated with further studies and its effect on the transition to the proerythroblast stage should be investigated.

The patients included in this study, harboring *RPS19* (two patients) and *CECR1* (one patient) mutations, presented with similar clinical features. This condition, defined as phenocopy, is also observed in the transcriptomic profile of proerythroblasts. At the beginning of the study, our expectation was to find differences in the transcriptomic signatures between the patient with *CECR1* mutation and those with *RPS19*. However, both the cellular stress-related gene expression pattern and the decrease in ribosomal protein gene expression showed a similar response or defect in all patients’ proerythroblasts. The increase in *BAZ2A* (responsible for silencing rRNA transcription) expression, in addition to the decrease in ribosomal protein gene expression, suggested that there may be a common inhibition in the ribosome synthesis pathway. In other words, while ribosomal protein synthesis was decreasing, ribosomal RNA transcription was also suppressed. Despite not being direct evidence, this observation suggested a specific cellular response rather than a secondary finding. The decrease in total ribosomal protein gene expression in cells with the *RPS19* mutation has been previously observed, but it has been an important finding that this condition is also seen in cells with the *CECR1* mutation (Morgado-Palacin et al., 2015).

The results of our study also suggested that the gene expression profile reflecting cellular stress and cytokine response in proerythroblasts may be associated with increased inflammation in the BM microenvironment. It has been shown that TNF-α inhibits erythroid differentiation (Buck et al., 2008). Recently, ribosomal stress-induced TNF-α production of non-erythroid cells and increase in p53 expression was reported in DBA patients (Bibikova et al., 2014). An increase in TNF-α production has also been demonstrated in individuals with *CECR1* mutation (Caorsi et al., 2017; Barzaghi et al., 2018). Therefore, the similar gene expression pattern in different patients with *RPS19* and *CECR1* mutations in the present study may suggest a contribution of TNF-α in an inflamed BM niche. It may also be a possibility that the inflammatory microenvironment decreases the expression of ribosomal proteins by causing cellular stress. Morgado-Palacin et al. (2015) previously demonstrated reduced ribosomal protein gene expression in *Rpl11* mutant mice. We also found a global decrease in ribosomal protein gene expression in proerythroblasts. However, the effect of *CECR1* mutations in DBA pathogenesis should be investigated in detail if there is a direct relationship with ribosome biogenesis.

When we focused on transcription factors, which are cardinal players of gene expression changes, we found evidence that may be directly related to the differentiation defect of erythroid lineage cells. *GATA1* and *GATA2* balance have critical importance in erythroid differentiation. At early stages *GATA1* expression is low, but increases as the process progress. Reversely, *GATA2* decreases during erythroid differentiation (Ferreira et al., 2005; Suzuki et al., 2011, 2013). We observed that *GATA1* and *GATA2* were up-regulated in DBA proerythroblasts compared to healthy donors. Moreover, we found increased expression of *KLF13* and *SKI*, negative regulators of erythroid differentiation (Ueki et al., 2004; Mitsuma et al., 2005; Singbrant et al., 2014). FOXO3 (forkhead box O3) is also one of the critical regulators of erythroid differentiation and regulates other gene expressions as well (Liang and Ghaffari, 2016). Therefore, the differential expression of *FOXO3* could lead to a misregulation in further pathways. Erythropoiesis is regulated by hypoxic conditions. At low oxygen levels, *ARNT* (aryl hydrocarbon receptor nuclear translocator, HIF-1ß) activates other cascades in order to maintain oxygen homeostasis (Liang and Ghaffari, 2016). Hence, the increase of *ARNT* expression may be the cause or consequence of the reduced oxygen-carrying mechanism. As mentioned in a previous study, *STAT3* (Signal Transducer and Activator of Transcription 3) is responsible for organizing a “fine-tune” EPOR (Erythropoietin Receptor) response (Mauracher et al., 2020). In addition, a gain of function mutation of *STAT3* leads to impaired erythropoiesis and anemia phenotype, also autoimmunity (Milner et al., 2015; Mauracher et al., 2020). Although it is not possible to determine the cumulative effect of all these changes, it can be speculated that DBA proerythroblasts have an imbalanced pattern in terms of transcription factor networks. This expression pattern could be an indicator/promoter of altered homeostasis.

According to the transcriptomic signature of the erythroid progenitor cells from the patient’s BM, we could speculate that inflammation is a critical component of DBA and this inflammation may cause erythroid failure. The reason that *CECR1* mutations have similar gene expression pattern with DBA is that erythroid lineage cells affect is the same way due to the inflammatory marrow microenvironment. Pesciotta et al. (2015) also stated that DBA patient samples showed an inflammatory signature in proteomics analysis and stated that DBA patients may have changes in BM microenvironment. Interestingly, Danilova et al. emphasized the role of immune system in DBA, mentioning the possible cause of DBA is activated immune system due to different cellular responses (Danilova et al., 2018).

In our study, DBA patient samples showed diminished or lack of colony-forming potential as expected. Two samples with *RPS19* are failed to form any colonies. However, interestingly, the sample with *CECR1* mutation formed a reduced number of colonies that have a similar morphology (and transcriptomic profile) with healthy donor samples. From a transcriptomic point of view, the gene expression profiles of the colonies did not show a significant difference, however, as also mentioned before, the transcriptomic profiles of BM -derived proerythroblasts of DBA patients were different. This data emphasizes the importance of using and analyzing pure patient-derived cells without any cultural treatment.

On the other hand, the possibility of the effect of steroid use and transfusion on the transcriptomic profile in BM cells used in this study should be kept in mind. The most important difference in this respect is that the patients involved in our study are under treatment and anemia phenotype is present. Analyzing more patients will be the most important factor in understanding both whether there are specific effects on treatment responses and whether patients with different genotypes will show similar expression patterns. The question that there may be sub-clinical inflammation in DBA patients should be answered in further studies.

Lastly, our findings regarding the BM resident cells may contribute to a better understanding of the cellular characteristics of DBA. Future studies investigating the effects of the inflammatory niche on cells in different stages of erythroid lineage might provide novel insights into this subject.

## Data Availability Statement

The datasets presented in this study can be found in Dryad Digital Repository, doi: 10.5061/dryad.3bk3j9kjg.

## Ethics Statement

The studies involving human participants were reviewed and approved by the Hacettepe University Non-interventional Clinical Researches Ethics Board. Written informed consent to participate in this study was provided by the participants’ legal guardian/next of kin.

## Author Contributions

BK, MK, GE, and ET performed the experiments and analyzed the data. ET and SU designed the research. FG, DU, and SU provided the patient samples. BK and ET wrote the manuscript. All authors corrected the manuscript, contributed to the article, and approved the submitted version.

## Conflict of Interest

The authors declare that the research was conducted in the absence of any commercial or financial relationships that could be construed as a potential conflict of interest.

## Footnotes

1 http://bioinformatics.sdstate.edu/idep/

2 https://biit.cs.ut.ee/gprofiler/gost

3 www.graphpad.com

4 https://datadryad.org/stash
