# Supplementary material for: Proerythroblast Cells of Diamond-Blackfan Anemia Patients With RPS19 and CECR1 Mutations Have Similar Transcriptomic Signature
Source: Front Physiol. 2021 Jun 11;12:679919. doi: 10.3389/fphys.2021.679919 (PMC8226250; doi:10.3389/fphys.2021.679919)
Supplement: Supplementary file 1 [file Data_Sheet_1.PDF]

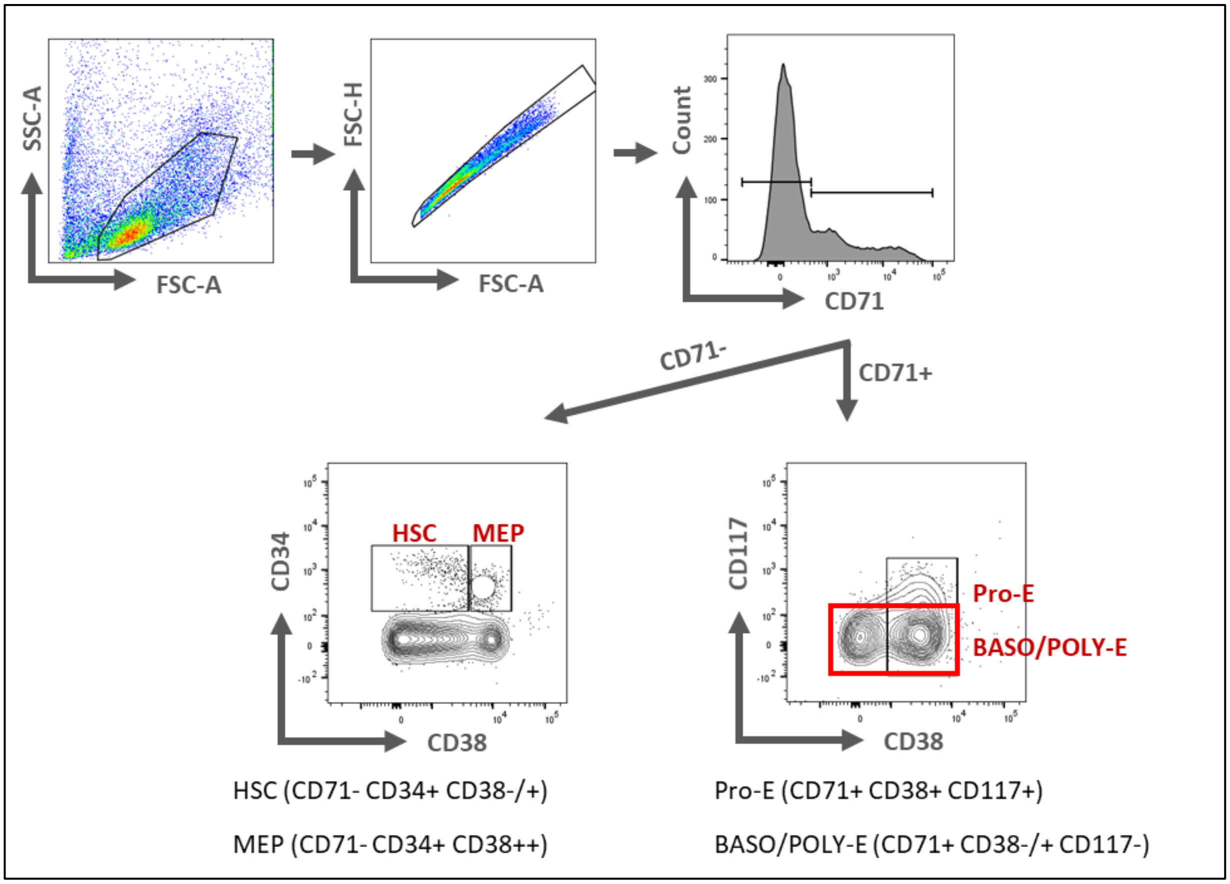

Supplementary Figure 1. Gating strategy for immunophenotyping and cell sorting. HSC: Hematopoietic Stem Cell (HSC), MEP: Megakaryocyte–Erythroid Progenitor Cell, Pro-E: Proerythroblast, Baso/Poly-E: Basophilic and Polychromatophilic Erythroblasts.
