# Supplementary material for: Proerythroblast Cells of Diamond-Blackfan Anemia Patients With RPS19 and CECR1 Mutations Have Similar Transcriptomic Signature
Source: Front Physiol. 2021 Jun 11;12:679919. doi: 10.3389/fphys.2021.679919 (PMC8226250; doi:10.3389/fphys.2021.679919)
Supplement: Supplementary file 2 [file Data_Sheet_2.PDF]

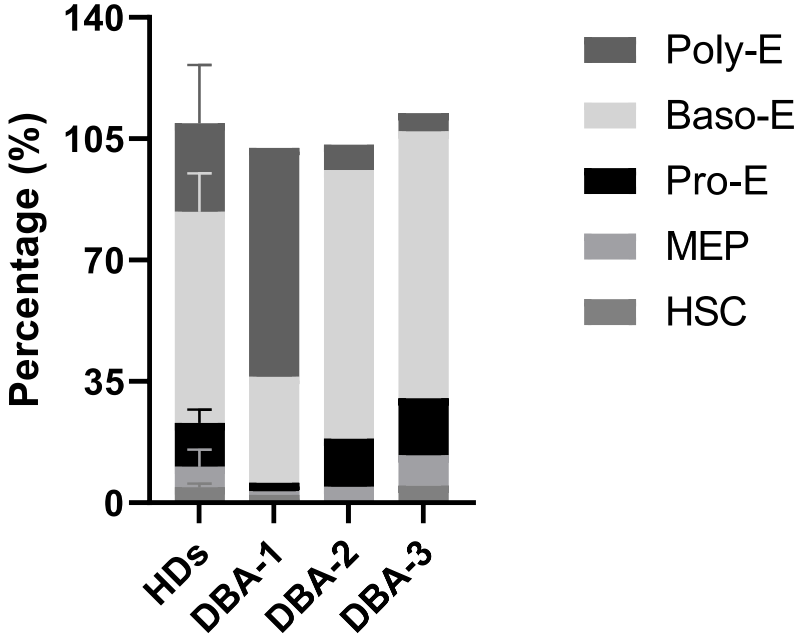

	HSC				MEP				Pro-E				Baso-E				Poly-E			
	HD-1	HD-2	HD-3	HD-4	HD-1	HD-2	HD-3	HD-4	HD-1	HD-2	HD-3	HD-4	HD-1	HD-2	HD-3	HD-4	HD-1	HD-2	HD-3	HD-4
**HDs**	4,85	5,63	3,24	4,2	9,64	14,1	3,53	1,4	11,8	9,1	11	20,3	77	57	50	67,3	7,96	32,2	48,4	10,2
**DBA-1**	2,25				1,06				2,51				30,5				66,1			
**DBA-2**	0,63				4,01				13,8				77,5				7,36			
**DBA-3**	4,93				8,82				16,4				77				5,2			

Supplementary Figure 2. Cell percentages after immunophenotyping and cell sorting. HSC: Hematopoietic Stem Cell (CD71- CD34+ CD38-/+), MEP: Megakaryocyte–Erythroid Progenitor Cell (CD71- CD34+ CD38++), Pro-E: Proerythroblast (CD71+ CD38+ CD117+), Baso-E: Basophilic Erythroblast, Poly-E: Polychromatophilic Erythroblast, HDs: Healthy Donors.
