# Supplementary material for: Proerythroblast Cells of Diamond-Blackfan Anemia Patients With RPS19 and CECR1 Mutations Have Similar Transcriptomic Signature
Source: Front Physiol. 2021 Jun 11;12:679919. doi: 10.3389/fphys.2021.679919 (PMC8226250; doi:10.3389/fphys.2021.679919)
Supplement: Supplementary file 4 [file Data_Sheet_4.PDF]

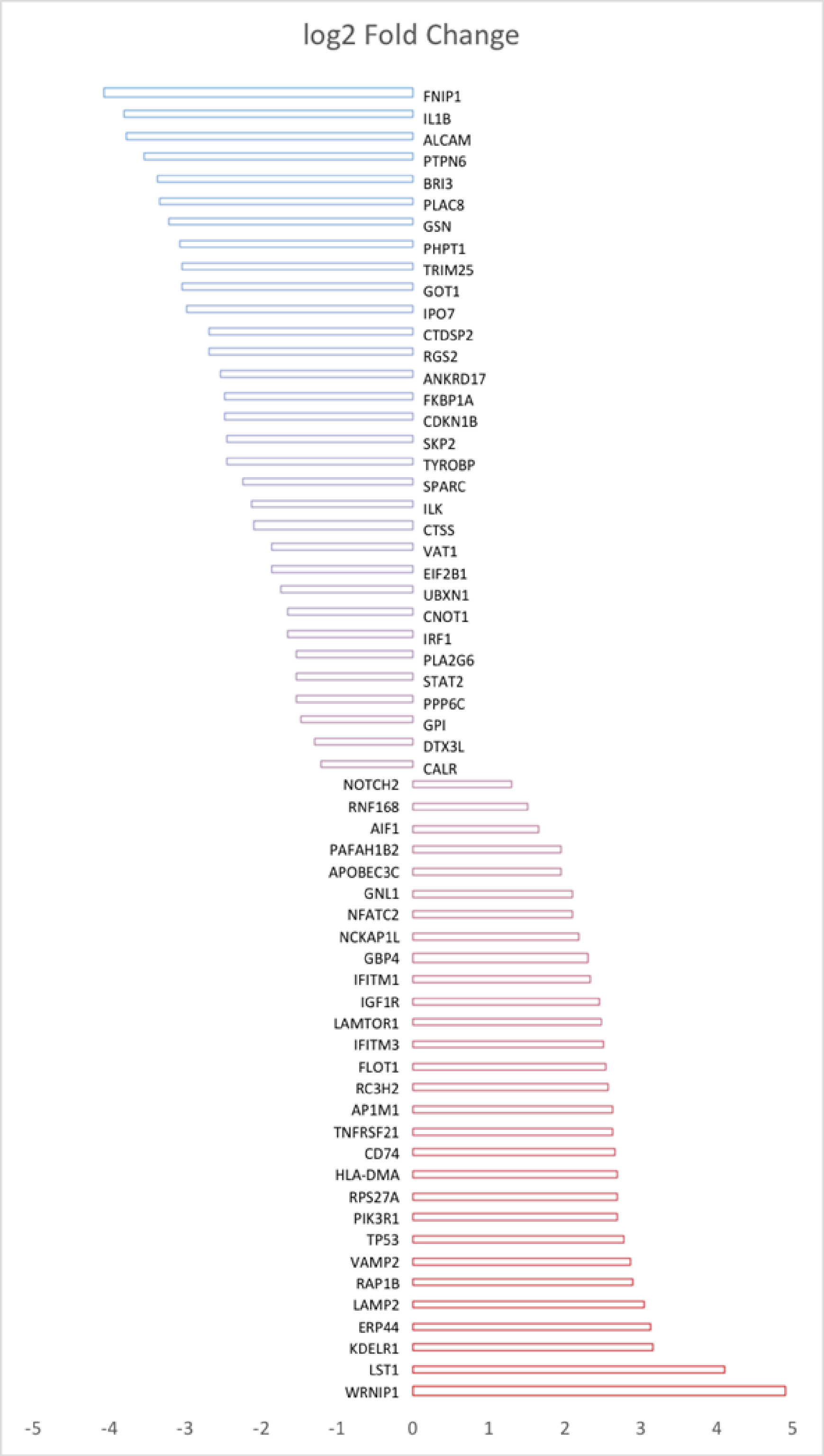

Supplementary Figure 4. Immune system related genes that are differentially expressed in DBA HSCs.
