# Supplementary material for: Proerythroblast Cells of Diamond-Blackfan Anemia Patients With RPS19 and CECR1 Mutations Have Similar Transcriptomic Signature
Source: Front Physiol. 2021 Jun 11;12:679919. doi: 10.3389/fphys.2021.679919 (PMC8226250; doi:10.3389/fphys.2021.679919)
Supplement: Supplementary file 5 [file Data_Sheet_5.PDF]

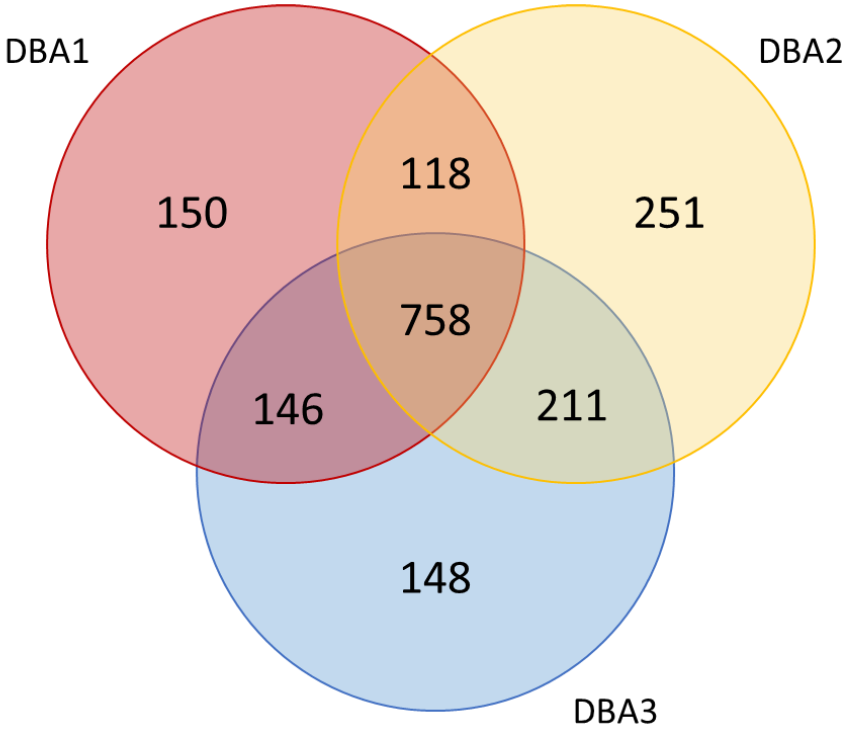

Supplementary Figure 5. Venn diagram of intersections between differentially expressed genes in each DBA patient. Differentially expressed gene numbers obtained by comparing proerythroblasts of healthy donors.
